# Supplementary material for: A Novel Orthotopic Liver Cancer Model for Creating a Human-like Tumor Microenvironment
Source: Cancers (Basel). 2021 Aug 8;13(16):3997. doi: 10.3390/cancers13163997 (PMC8394300; doi:10.3390/cancers13163997)
Supplement: Supplementary file 1 [file cancers-13-03997-s001.zip › cancers-1306316-supplementary.pdf]

Supplementary Materials

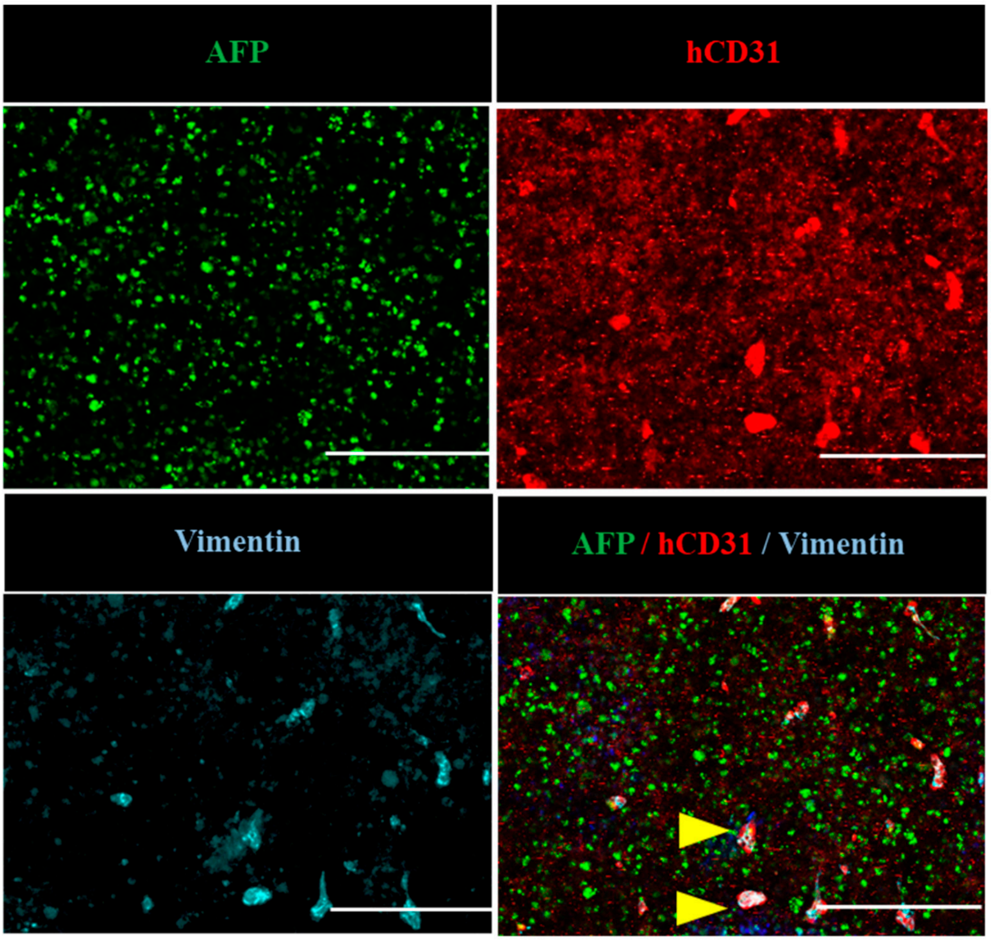

Figure S1. Immunofluorescence staining of AFP (green), hCD31 (red), Vimentin (light blue) in HCC organoid. The scale bar represents 500 µm.

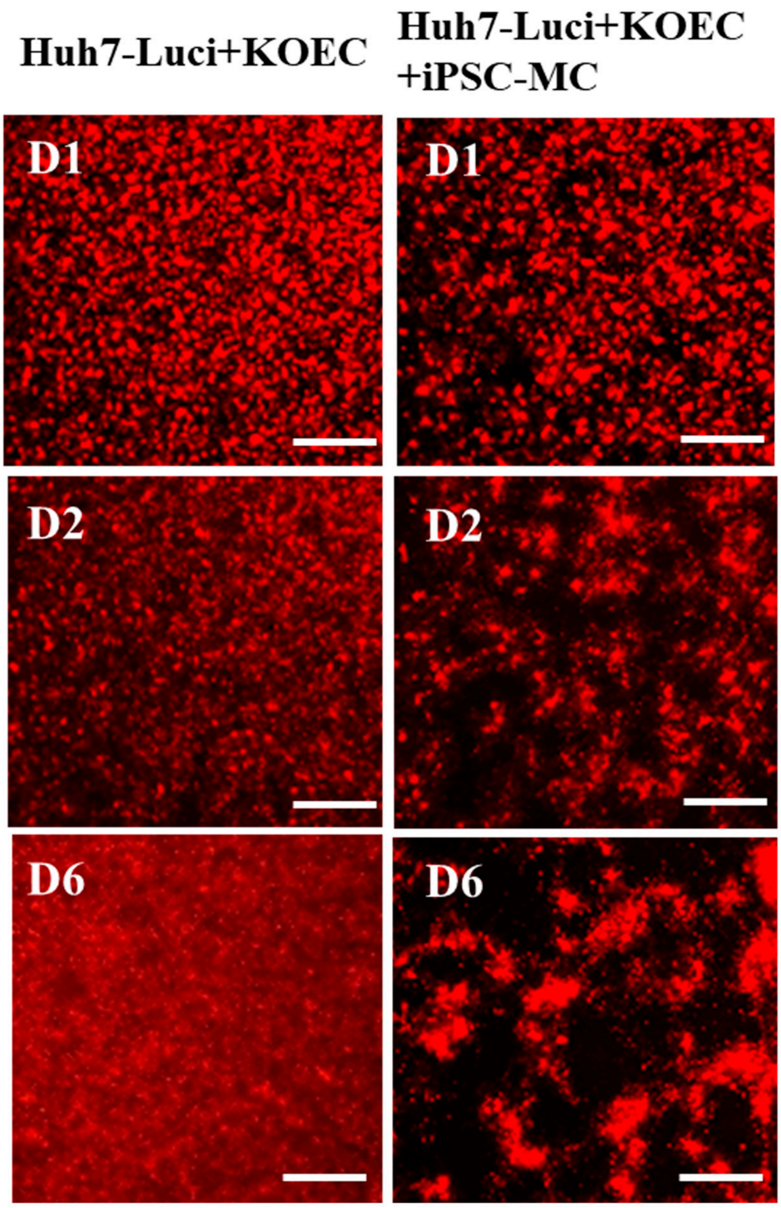

Figure S2. Live-cell imaging of KO-EC with Huh7-Luci and Huh7-Luci+iPSC-MC in the Ibidi Culture-Insert system at day 1, 2, 6. The scale bar represents 200 μm.

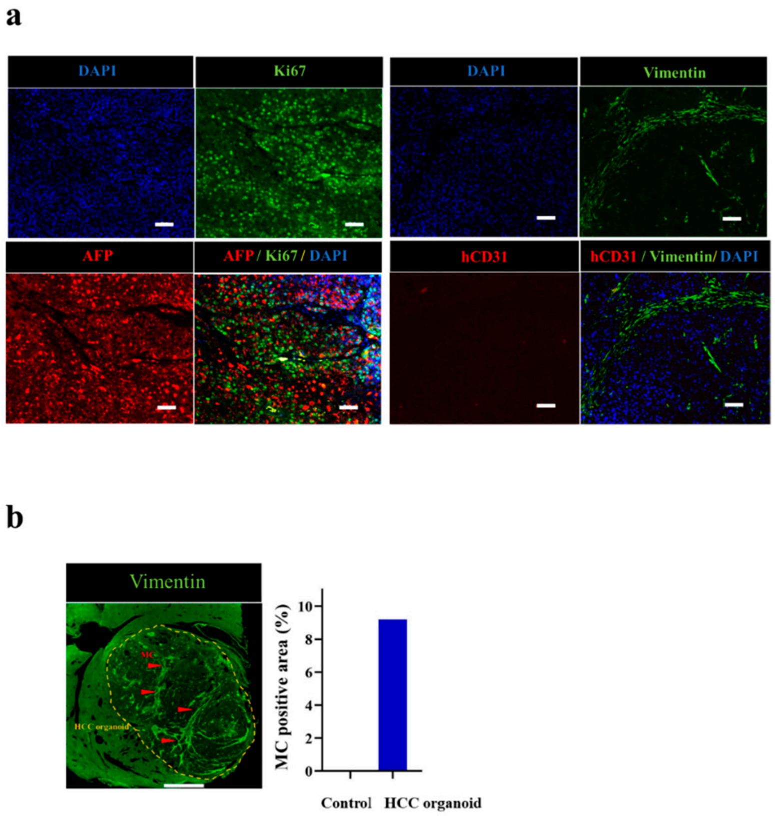

Figure S3. Immunofluorescence staining of HCC organoid in vivo. (**a**) Immunofluorescence staining of AFP (red), Ki67 (green), hCD31 (red), Vimentin (green), DAPI (blue) in HCC liver section. The scale bar represents 50 μm. (**b**) The image of MC area in immunofluorescence staining. The yellow dotted line indicates the HCC organoid. Red arrows indicate MC. HCC organoid (without iPSC-MC was used as control). The scale bar represents 0.1 mm.

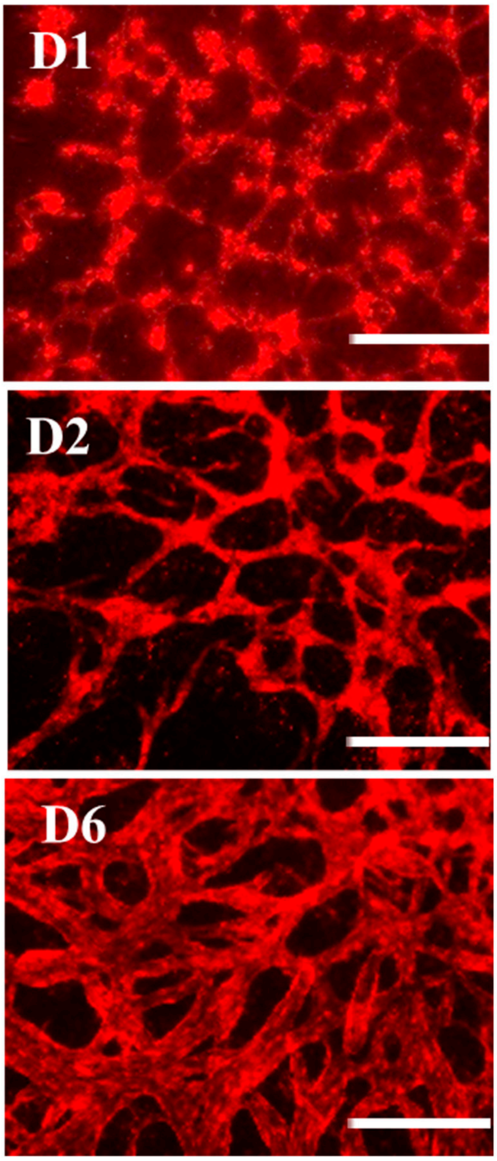

Figure S4. Live-cell imaging of hepatic endoderm with KOEC and iPSC-MC in the ibidi Culture-Insert 2 well system at day 1, 2, 6. The scale bar represents 500 μm.

Without UH

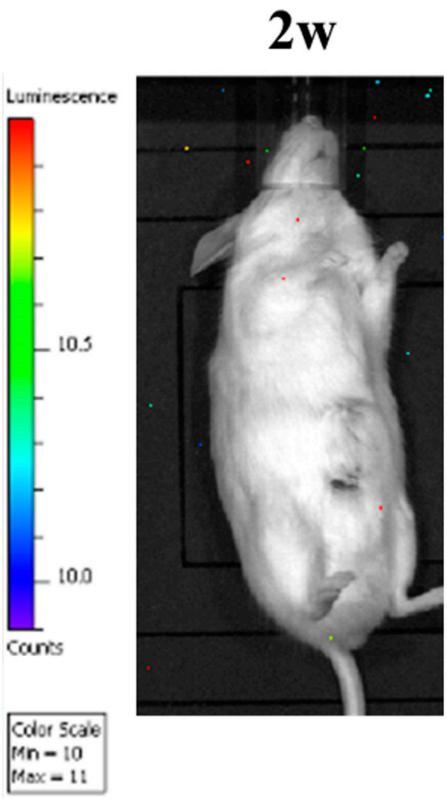

Figure S5. Non-invasive BLI of mouse implanted with HCC organoid without UH after 2 weeks, n = 4/group.

a

b

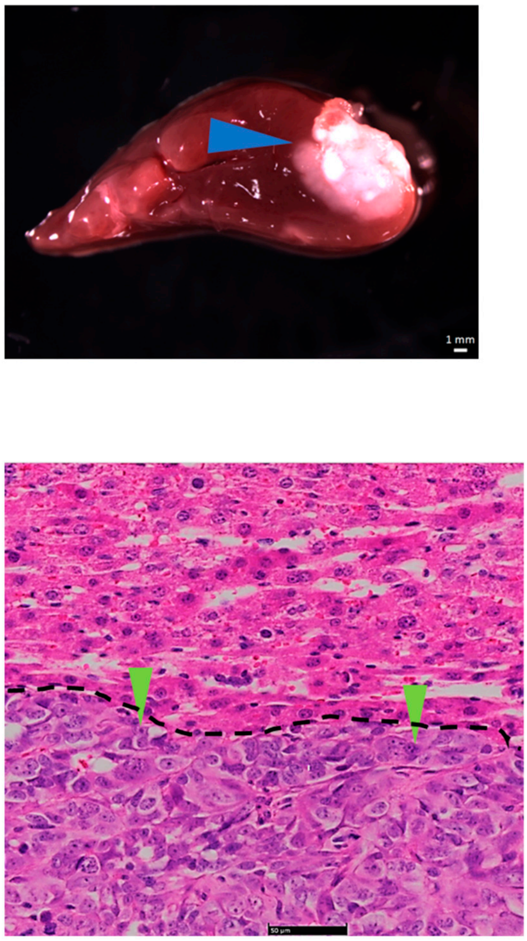

Figure S6. Implantation methods of human liver cancer (HepG2) organoid onto the mouse liver surface. (**a**) Morphology image of 2w after implantation of HepG2 organoid. Blue arrows indicate tumor tissue. The scale bar represents 1 mm. (**b**) The HE staining of mice normal liver and liver cancer. The black dotted line indicates the junction of normal liver tissue and tumor tissue. Green arrows indicate tumor cells. The scale bar represents 50 µm.

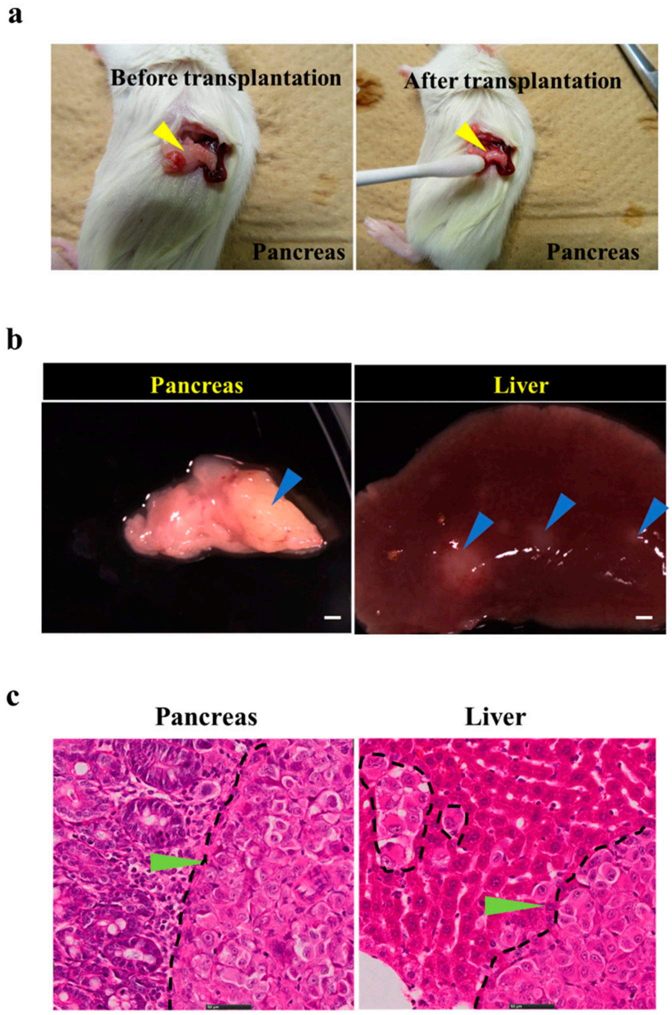

Figure S7. Implantation methods of human pancreas cancer (CFPAC-1) organoid onto the mouse pancreas body surface. (**a**) Images of the implantation procedure of pancreas cancer organoid over the mouse pancreas. Yellow arrows indicate pancreatic tissue. (**b**) Morphology image pancreatic organoid of 8w after implantation. Carcinoma in situ (left), distant metastasis liver lesions (right). Blue arrows indicate tumor tissue. The scale bar represents 2 mm. (**c**) The HE staining of mouse pancreas (**left**) and liver (**right**). Black dotted line indicates the junction of normal pancreatic tissue and tumor tissue. Green arrows indicate tumor cells. The scale bar represents 50 μm.
